# Investigation of Interaction between the Spike Protein of SARS-CoV-2 and ACE2-Expressing Cells Using an In Vitro Cell Capturing System

**DOI:** 10.1186/s12575-021-00153-9

**Published:** 2021-08-26

**Authors:** Yuning Shang, Feixiang Chen, Shasha Li, Lijuan Song, Yunzhen Gao, Xinhua Yu, Junfeng Zheng

**Affiliations:** 1 Institute of Psychiatry and Neuroscience, Xinxiang Medical University, XinXiang, China; 2 Priority Area Asthma & Allergy, Research Center Borstel, Airway Research Center North (ARCN), Members of the German Center for Lung Research (DZL), Borstel, Germany

**Keywords:** SARS-CoV-2;spike protein, ACE2, In vitro cell-capture

## Abstract

**Background:**

The Interaction between severe acute respiratory syndrome coronavirus 2 (SARS-CoV-2) spike protein with Angiotensin converting enzyme 2 (ACE2) on the host cells is a crucial step for the viral entry and infection. Therefore, investigating the molecular mechanism underlying the interaction is of great importance for the prevention of the infection of SARS-CoV-2. In this study, we aimed to establish a virus-free in vitro system to study the interaction between the spike protein and host cells of SARS-CoV-2.

**Results:**

Our results show that ACE2-overexpressing HEK293T cells are captured by immobilized spike S1 protein, and the cell capturing process can be inhibited by the receptor binding domain of the spike protein or antibodies against S protein. Furthermore, spike S1 protein variant with D614G mutant show a higher cell capturing ability than wild type spike S1 protein and stronger binding capacity of its receptor ACE2. In addition, the captured cells can be eluted as living cells for further investigation.

**Conclusions:**

This study provides a new in vitro system for investigating the interaction between SARS-CoV-2 and host cells and purifying ACE2-expressing cells.

**Supplementary Information:**

The online version contains supplementary material available at 10.1186/s12575-021-00153-9.

## Background

Angiotensin converting enzyme2 *(*ACE2) is a key cell entry receptor for severe acute respiratory syndrome coronavirus 2 (SARS-CoV-2), the causal agent of coronavirus disease 2019 (COVID-19) [1, 2]. Interaction between the spike (S) protein of the virus and the entry receptor on human airway epithelial cells is a crucial step for the viral infection [2, 3]. Therefore, investigating the molecular mechanism underlying the interaction is of great importance for the prevention of the infection of SARS-CoV-2. For that purpose, multiple in vitro experimental systems have been generated and utilized. Such in vitro systems usually consist of host cells and authentic and/or pseudo typed SARS-CoV-2 virus, which allows determining the efficiency of viral entry [3–6]. However, working with coronavirus requires a special, high-security laboratory, making it challenging to perform the research in ordinary laboratories. Although pseudovirus are widely used [7, 8], interaction between pseudovirus and host cells consists of multiple steps such as receptor binding, protease activation, membrane fusion, and viral entry [9], making it difficult to precisely examine a single factor, such as receptor binding. Also, accurate quantification of pseudovirus infection is a complicated and technically experience demanding system [10]). Therefore, a virus-free in vitro experimental system enabling to the investigation of the interaction between S proteins and host cells is of interest.

In this study, we aimed to develop an in vitro system to study the interaction of SARS-CoV-2 Spike S1 protein with ACE2-expressing cells. By using immobilized S protein of SARS-CoV-2, this in vitro system can specifically capture ACE2-expressing cells, and the captured cells can be eluted as living cells for further investigation. Therefore, this in vitro system can be utilized for the investigation of virus-host interaction, the evaluation of the ability to neutralize antibodies and antagonists, as well as the isolation and purification of host cells of SARS-CoV-2.

## Results

### Establishment of an In Vitro ACE2-Expressing Cell Capturing System

**Fig. 1:**
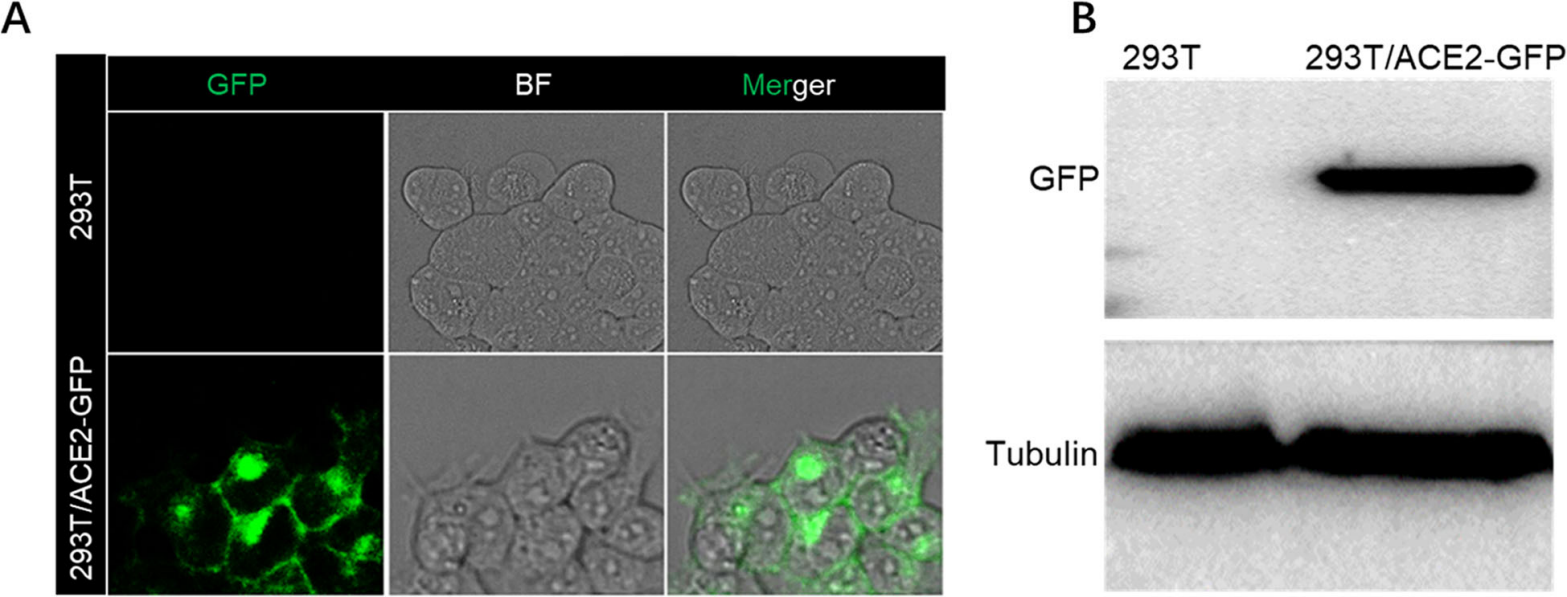
Construction of HEK293T cells line that continuously expressing ACE2-GFP. HEK293Tcells were transfected with pCMV-ACE2-GFPSark tag plasmid and selected with hygromycin B to generate cell lines expressingACE2-GFP, cells were passaged 10 times to ensure stable expression. **a** Confocal imaging of HEK293T cell line with stably expressed ACE2-GFP. **b** Cell lysates were analyzed by Western blot with antibodies specific for ACE2 to confirm the expression of ACE2-GFP in HEK293T cells

To establish an in vitro ACE2-expressing cell capturing system, we first generated a cell line that stably expresses human ACE2. For this purpose, HEK293T cells were transfected with an ACE2*-*GFP fusion plasmid, allowing the visualizing of the expression of ACE2. As shown in Fig. 1, ACE2-GFP could be detected on the surface of the transfected HEK293T, and the expression of ACE2 was further confirmed by western blotting.

**Fig. 2:**
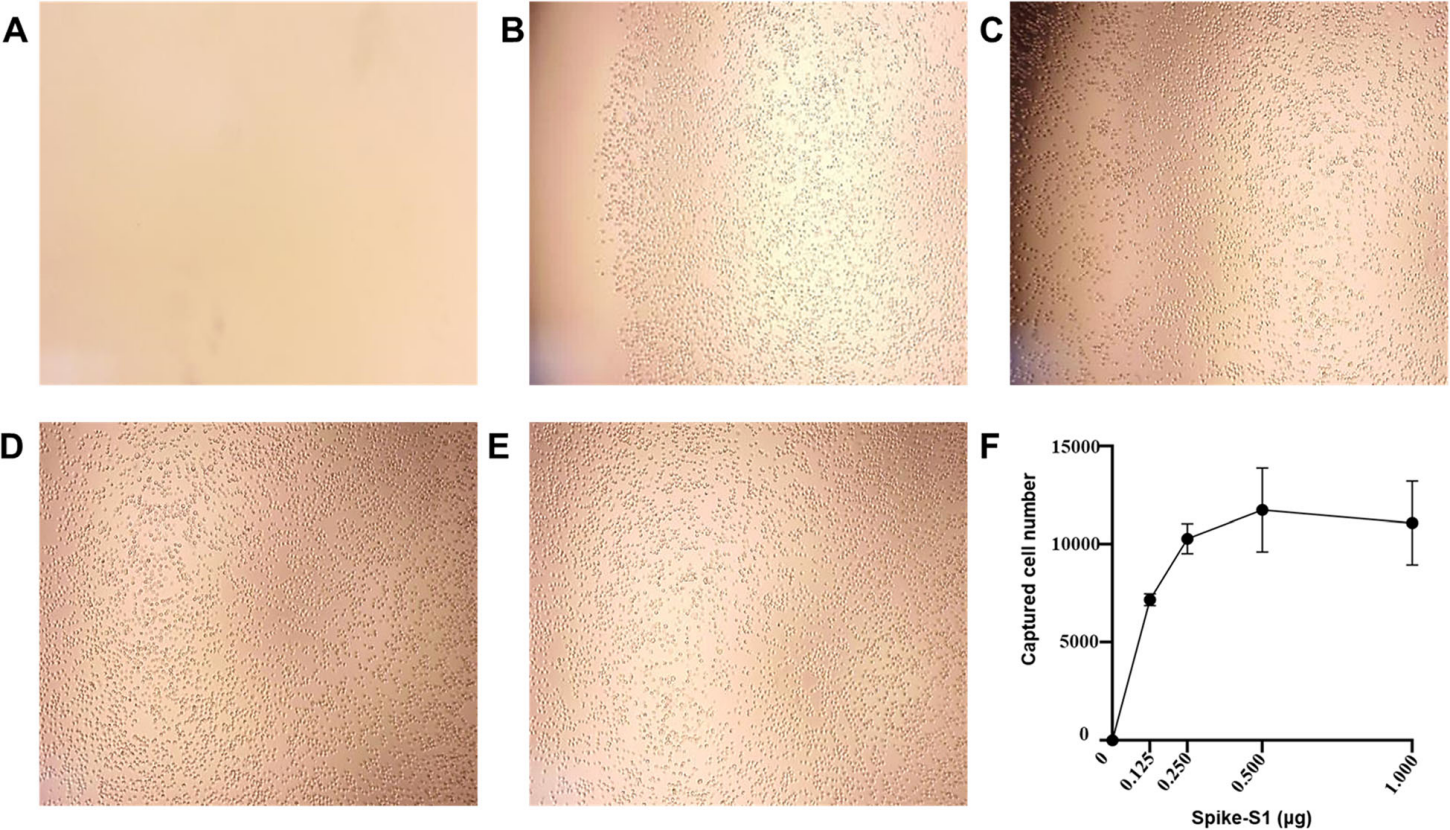
Establishment of the in vitro cell capturing system using immobilized spikeS1 protein. **a**-**e**, Optimization of the dose of immobilized spike S1 protein for capturing HEK293T/ACE2-GFP cells. Different amounts of S1 protein were coated on the 96-well microplate to capture the HEK293T/ACE2-GFP cells. Representative micrographs of captured cells are shown for 0 μg (A),0.125 μg(B),0.25 μg (C),0.5 μg(D),and 1.0 μg(E). **F** Quantification of captured cells using CCK8 test, data are presented as mean ± SD

In the next step, we investigated whether HEK293T/ACE2-GFP cells can be captured by immobilized Spike S1 protein of SARS-CoV-2. Different amounts of S1 protein ranging from 0 to 1 μg were immobilized on microplate surfaces, and HEK293T/ACE2-GFP cells captured by the S1 protein-coated surface were quantified using the CCK8 test. As shown in Fig. 2, the number of HEK293T/ACE2-GFP cells captured by S1 protein-coated surface increased accordingly with the increase of immobilized S protein, and it reached a plateau when the coated proteins were higher than 0.5 μg. By contrast, no HEK293T/ACE2-GFP cell was captured by uncoated surface. To better characterize the in vitro system, we determined the time kinetics of the cell capturing process. Captured HEK293T/ACE2-GFP cells were detectable 5 min after the incubation, and the number of captured cells increased with the incubation period and reached a relatively stable level when the incubation period was more than 45 min (Supplementary figure 1).

**Fig. 3:**
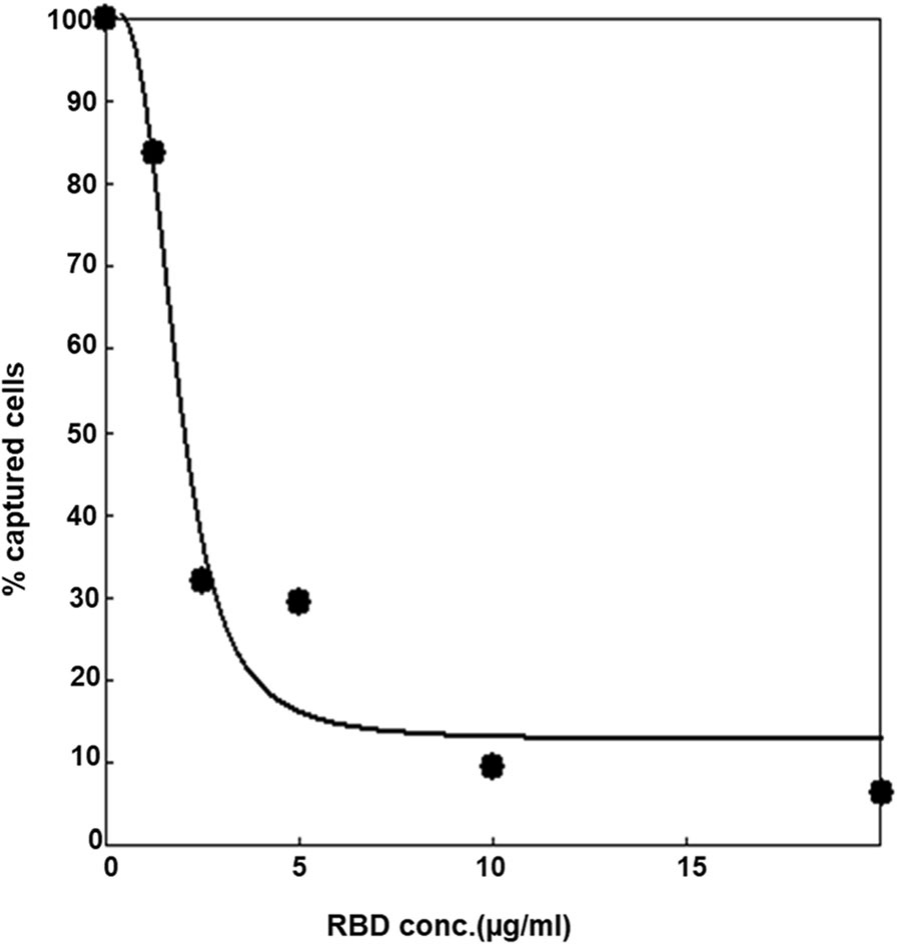
Competitive curve of cell capturing inhibition by RBD domain of Spike protein. 0.5 μg Spike S1 protein was coated on the microplate to capture the HEK293T/ACE2-GFP cells in presence of different concentrations of spike RBD protein. Amount of captured cells were determined by CCK8 test and expressed as relative value by setting the capability to capture cells of the non-RBD group as 100%. Data were plotted with a four-parameter logistic (4PL) regression curve fit of relative cell number (y-axis) versus the competitor concentration (x-axis)

To determine the specificity of the in vitro system, we utilized the receptor-binding domain (RBD) of S protein which is critical for the recognition of ACE2 to inhibit the cell capturing process. As shown in Fig. 3, RBD could efficiently inhibit the cell capturing process, with maximal inhibition of approximately 90% and half-maximal inhibitory concentration (IC50) of 2.0 μg/ml.

**Fig. 4:**
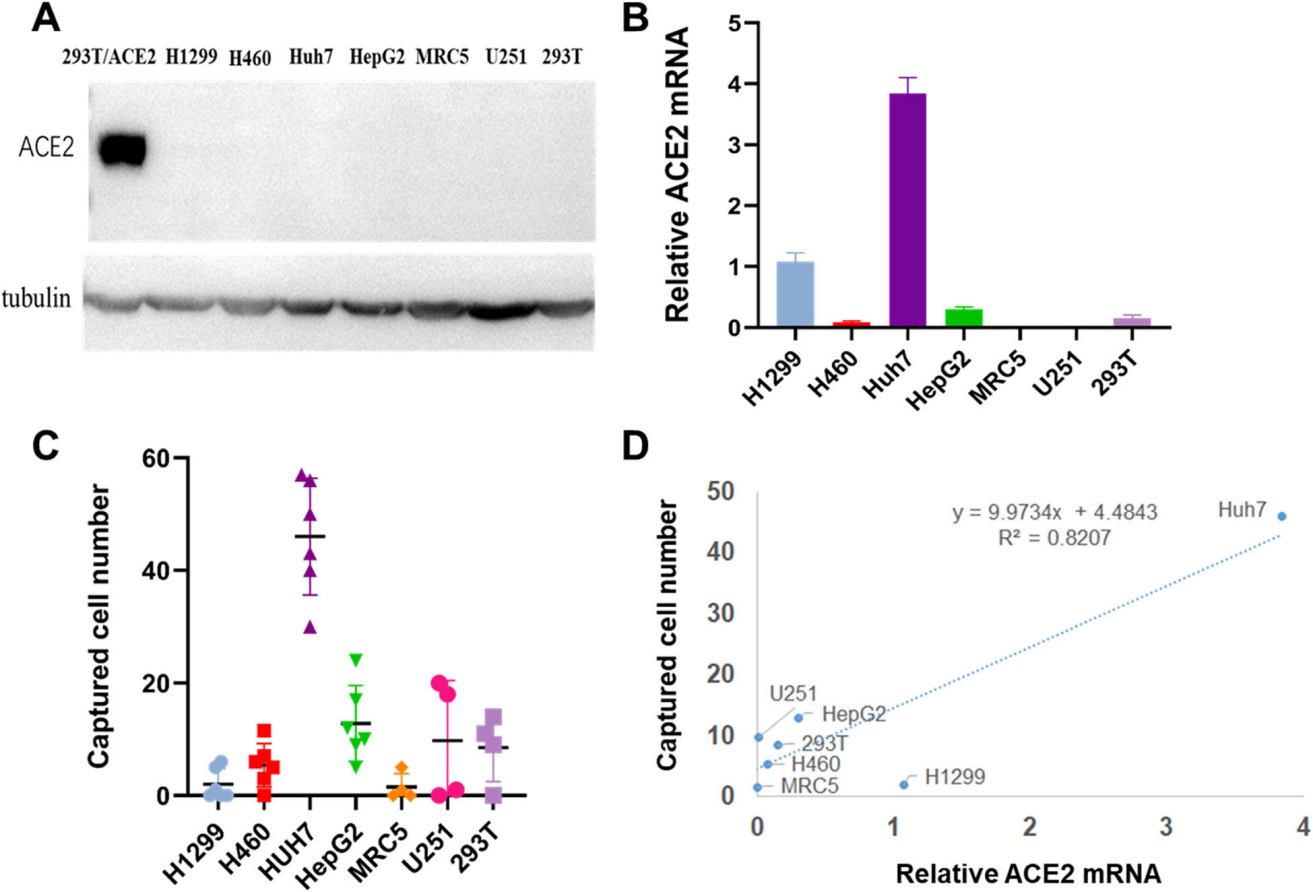
Correlation between expression of ACE2 on target cells and cell capturing ability. Endogenous expression of ACE2 in different cell lines determined by **A** Western blotting and **B** qPCR. **C** The cell capture ability of immobilized S1 to different cell lines. Captured cells were counted manually under the optical microscope; **D** correlation between ACE2 RNA level and S1 captured cell number fit a linear relationship with R^2^ = 0.82

Since HEK293T/ACE2-GFP cells overexpress ACE2, we next investigated cells without exogenous ACE2 expression can be captured by immobilized S protein of SARS-CoV-2. Seven cell lines were utilized in this experiment, including H1299, H460, HUH7, HepG, MRC5, U251 and HEK293T. We first determine the expression of ACE2 in those cells at both protein and mRNA levels. As shown in Fig. 4A, expression of ACE2 HEK293T/ACE2-GFP cells could be detected using western blotting, while levels of ACE2 in H1299, H460, HUH7, HepG, MRC5, U251 and HEK293T were under the detection limit. We then determined the expression of ACE2 at mRNA level using quantitative PCR. Among the seven cell lines, Huh7 showed the highest expression levels of ACE2, followed by H1299, HepG2 (Fig. 4B). Accordingly, the number of captured Huh7 cells also was higher than that of the other six cell lines (Fig. 4C). Also, the number of captured cells was highly correlated to the expression of ACE2 genes (R2 = 0.82, Fig. 4D).

Taken together, ACE2-expressing cells could be specifically captured by immobilized S1 protein, providing an in vitro system for investigating the interaction between S protein of SARS-CoV-2 and host cells.

### Application of the In Vitro Cell Capturing System

**Fig. 5:**
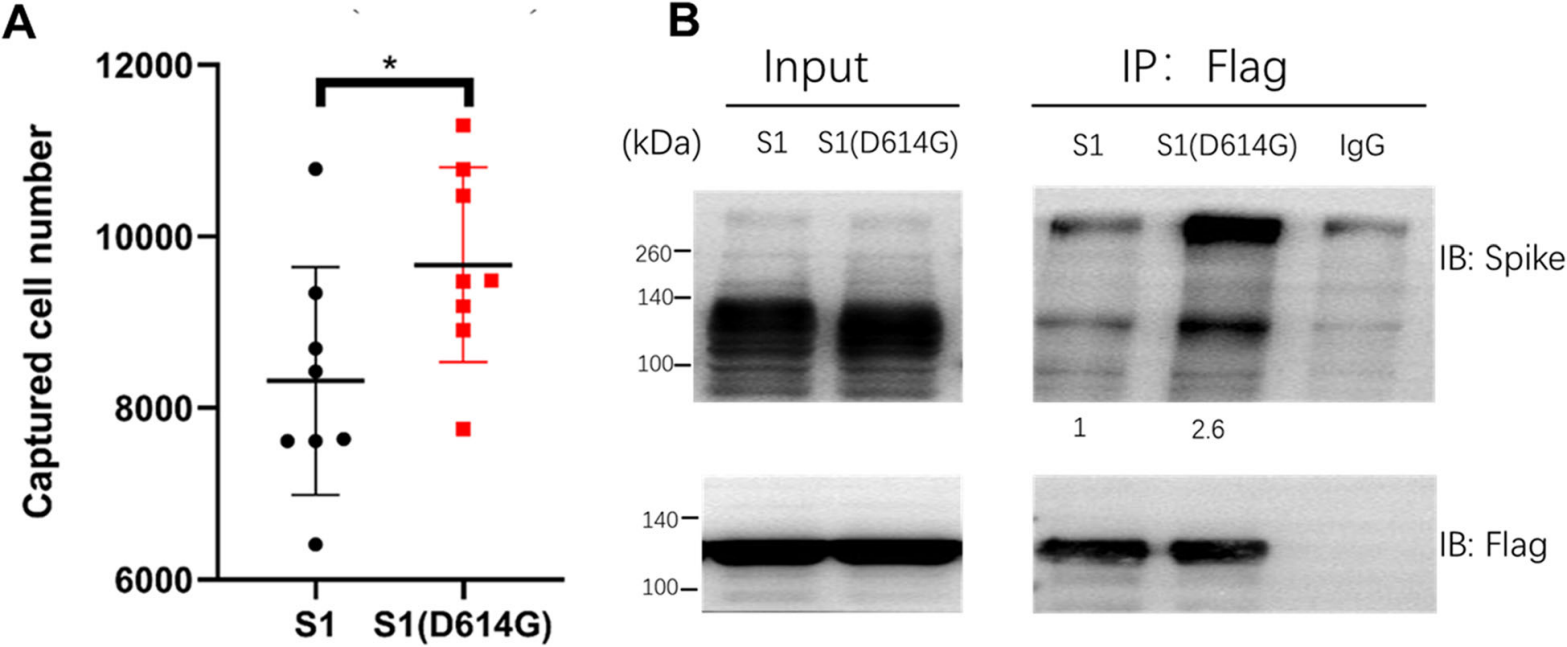
The D614G mutation increases ACE2-expressing cell capturing ability of S protein. **A** S1 or D614G S1 variant was immobilized on 96 well microplates, and HEK293 cells stably expressing ACE2-GFP were incubated with ACE2-293 T cells. Captured cells were detached by Trypsin and counted by CCK8 test. Quantitation data are presented as mean ± SD; *** *p* < 0.05 by unpaired Student t-test. **B** FLAG-tagged ACE2 stable expressed cells lysate were incubate with Spike S1 or S1(D614G) proteins. After FLAG-IP, the immunocomplexes were blotted with anti-FLAG or anti-spike antibodies as indicated. Spike S1 monomer (apparent molecular weight ~ 116Kd) and trimer (apparent molecular weight ~ 300Kd) were found after the western blotting. Fluorescence intensity quantification of S1 or S1(D614G) indicated below each band were normalized by each input.

A potential application of this in vitro cell capturing system is evaluating the binding ability of mutant S protein variants to ACE2-expressing cells. To access this possibility, we compared the cell capturing ability of wild type S1 protein and S1 protein with the amino acid change from aspartate to a glycine residue at position 614(D614G) which is associated with enhanced infectivity and increased spike-ACE2-binding affinity [11, 12]. As shown in Fig. 5A, the S1 protein with D614G mutation showed a significantly higher cell capturing ability than wild type S protein. We further determined whether the binding affinity between S1(D614G) and ACE2 is more stronger by a Co-IP experiment. As shown in Fig. 5B, incubated with S1 / S1(D614G) proteins and ACE2-expresing cell lysate, then pull down the ACE2-S1 protein complex with antibodies. We found that S1(D614G) bind more ACE2 proteins. This data prove that higher cell capturing ability of S1(D614G) is due to its stronger affinity to ACE2.

**Fig. 6:**
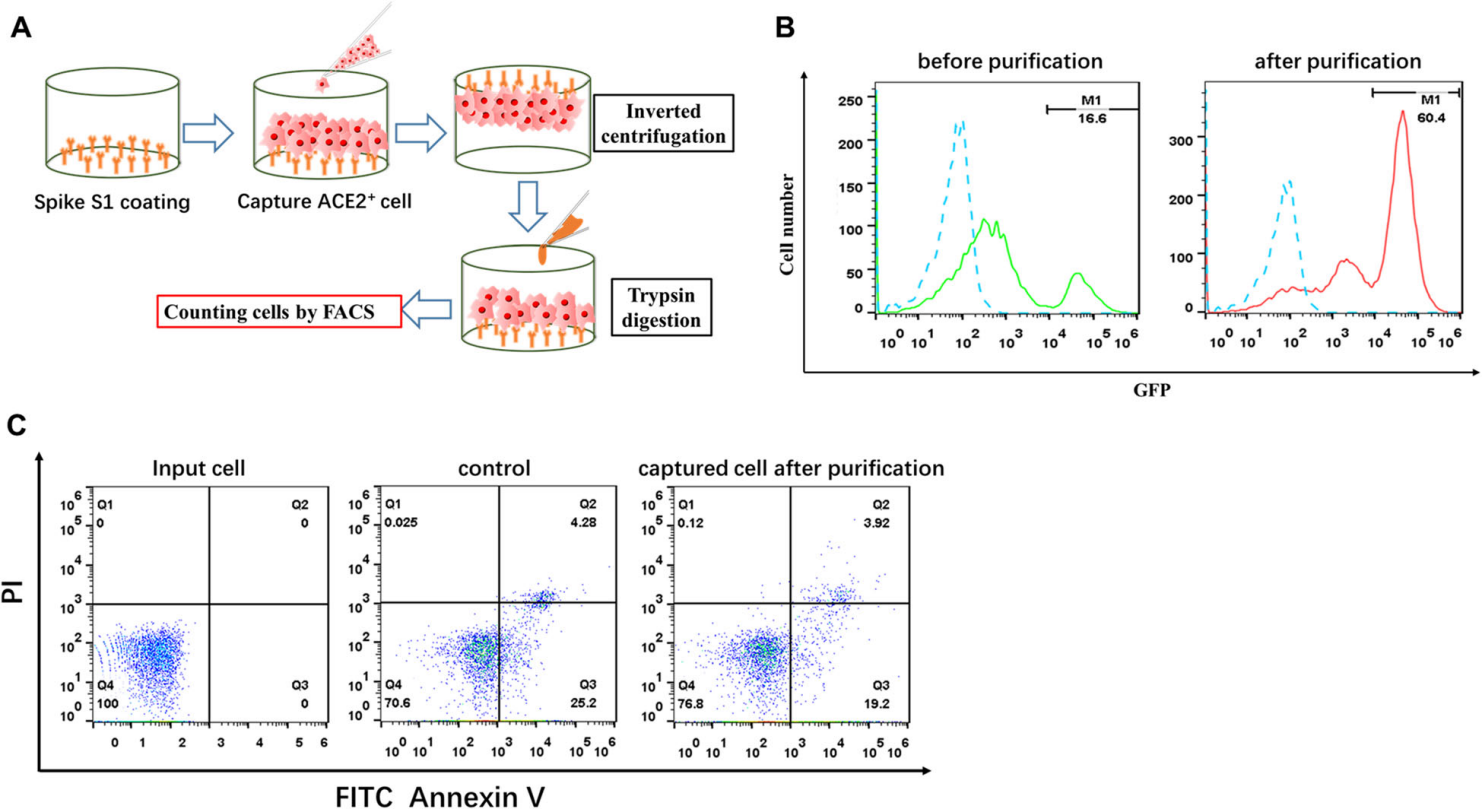
Detachment and purification of captured cells. **A** A schematic overview of a 2-step cell purification protocol. In the first step, captured cells were invertedly centrifuged to remove the weakly-bound cell. In the second step, remaining cells were detached by treatment of trypsin. **B** Expression of ACE2 on transiently transfected ACE2-293 T cells before (left panel) and after (right panel) purification and gate M1 indicates the proportion of cells with high expression of ACE2-GFP. **C** Cell viability of ACE2-293 T cells before and after the purification. Cell viability was determined using Annexin-V/PI staining

Another potential application of the immobilized S protein-based cell capturing system is isolating and purifying host cells of SARS-CoV-2. We first tested whether this in vitro system can be used to purified ACE2-expressing cells. For this purpose, HEK293T cells transiently transfected with ACE2-GFP were captured by immobilized S protein and then purified with a 2-step elution method (Fig. 6A). In this 2-step elution protocol, firstly captured cells were invertedly centrifuged to remove the weakly-bound cell and then eluted by treatment of trypsin. Compared with cells before the purification, cells eluted with the 2-step protocol contained a drastically higher percentage of cells that expressed higher levels of ACE2 (Fig. 6C). Next, we first determined whether the captured cells can be collected for further investigation. Captured cells were eluted after treatment with trypsin and accessed for cell viability. As shown in Fig. 6C, the majority of eluted cells were living cells, and cell viability was comparable between cells before and after the process of purification, In addition, the eluted cells were able to be further cultured and recaptured by S1 protein (data not shown). This finding suggests that cells with high binding affinity to S protein can be separated from those with low binding affinity.

**Fig. 7:**
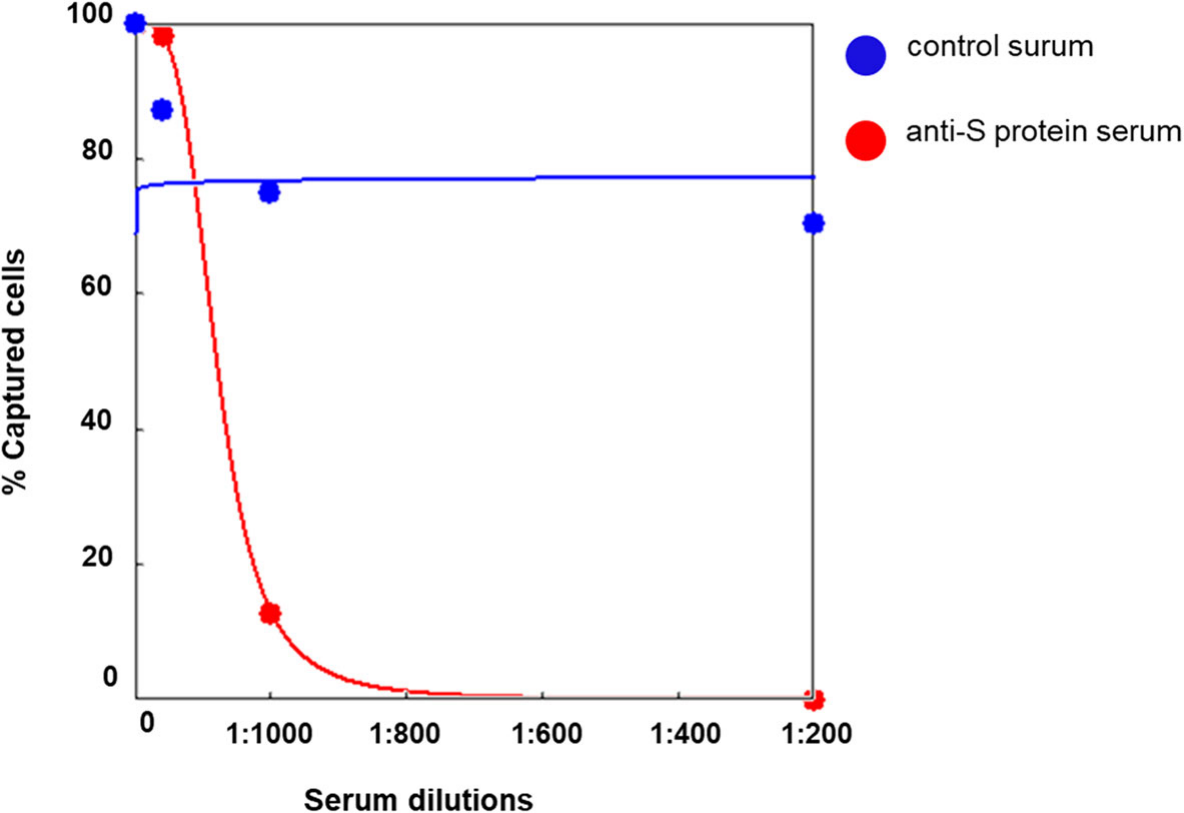
Competitive curve of cell capturing inhibition by serum purified from Spike-protein-immunized mice. Serum from S-protein immunized non-immunized mice were titrated and pre-incubated in SARS-CoV-2 Spike S1 coated wells for 1 h before the addition of ACE2-HEK293 cells. Data were plotted with a four-parameter logistic (4PL) regression curve fit of the relative amount of captured cells determined by CCK8 test (y-axis) versus 1:2n diluted serum samples (x-axis)

The third potential application of this in vitro system is to identify neutralizing antibodies or screen inhibitors. To test this potential, we generated polyclonal antibodies against S1 protein in B6 mice and determined whether the polyclonal antibodies can block the cell capturing ability of immobilizing S1 protein. As shown in Fig. 7, 1:200 diluted serum from mice immunized with S1 protein completely inhibited the cell capturing process, while control murine serum did not.

Therefore, these results demonstrated that this in vitro cell capturing system is useful for determining neutralizing antibodies.

## Discussion

RNA viruses are featured by a high mutation rate which is correlated with virulence modulation and evolvability, traits considered beneficial for viral adaptation [13]. During the transmission among humans and animals, SARS-CoV-2 is steadily mutating and many non-synonymous mutations have been reported [11]. Those mutations might change the binding affinity of SARS-CoV-2 to host cells, and thus will affect the infection efficiency and viral transmission, e.g. the D614G mutation [14]. Using the in vitro cell capturing system, we showed that S protein variant with D614G mutation showed a higher cell capturing ability than wild type one, which is in line with its effect on infection efficiency and binding affinity [7, 8, 11, 12, 14]. Thus, this in vitro experimental system provides an additional method to evaluate the effect of mutant S proteins of SARS-CoV-2. Besides, the cell capturing ability of immobilized S proteins could be inhibited by soluble RBD of S protein, suggesting that this in vitro assay can be neutralized. Given that anti-S protein neutralizing antibodies targeting SARS-CoV-2 spike (S) block the viral entry into cells via ACE2 [15], this in vitro cell capturing system is potentially useful for screening neutralizing antibodies. However, this notion needs to be verified experimentally.

Besides, our results showed that captured host cells can be eluted as living cells which can be used for further investigation. This feature is useful to discover potential spike protein receptors besides ACE2 and has certain advantages over pseudovirus methods. The feature of our cell capturing system is a one-step affinity separation of receptor-containing cells which is better than pseudovirus which contains complex progress including receptor binding, membrane fusion, and endocytosis [16, 17]. It is difficult to tell the precise role of the binding of the S1 protein to the receptor in a pseudovirus infection experiment. Second, to obtain Spike binding cells, after pseudovirus infection the target cells still need to be separated and purified from uninfected cells, which is time-consuming and purity limited in later RNA-seq or Mass spectrometry analysis.

## Conclusions

By using immobilized S proteins, this study showed that ACE2-expressing cells could be captured, and this cell capturing process can be specifically inhibited by adding the soluble RBD of S protein or anti-S protein antibodies. Therefore, this study provides a virus-free in vitro experimental system for studying the interaction between S protein of SARS-CoV-2 and their host cells.

## Materials and Methods

### Cell Culture

ALL cell lines used in this study were purchased from the Cell Bank of the Chinese Academy of Sciences (Shanghai, China). HEK293T (human, kidney), Huh-7 (human, liver), MRC-5 (human, lung), U251M (human, brain) cells were cultured in DMEM (Dulbecco’s’ modified Eagle medium) (Hyclone), H1299 AND H460 (human, lung) cells were cultured in RPMI-1640(Hyclone), MRC5(human, lung) and HepG2 (human, lung) cells were cultured in MEM (Minimum Essential Medium, Gibco) at 37 °C and 5% CO_2_ in a humidified atmosphere, supplemented with 10% fetal bovine serum (FBS) and penicillin (100 μg/ml), streptomycin sulfate (100 μg/ ml), 1x non-essential amino acid solution (10x stock, PAA) and 10 mM sodium pyruvate (ThermoFisher Scientific). For normal seeding and subcultivation, cells were first washed with phosphate buffered saline (PBS) and then incubated in the presence of trypsin/EDTA solution (Cytiva) until the cells detached. For capturing, cells were detached with Enzyme-Free Cell Dissociation Solution (ThermoFisher).

### Plasmids

Expression plasmids ofSARS-CoV-2 spike (QHD43416.1) and S1 with or without C-terminal HA epitope tag were purchased from Sino Biological. human ACE2 tagged with GFP (ACE2-GFP) was purchased from Sino Biological (HG10108-ACG).

### Construction of Cell Line with Stable ACE2 Expression

HEK293T cells were transfected with pCMV-ACE2-GFPSark tag expression plasmid by lipo3000 according to manual procedure, Hygromycin B selected (110 μg/ml) for 7d and analyzed for ACE2-GFP expression by Western blot and immunostaining using an antibody to ACE2 (ACE2–10108-T24, Sino Biological).

### qRT-PCR Analysis

Total RNA of 293 T, Huh7, HepG2, H1299, H460, U251 and MRC5 cells were isolated using TransZol Up plus RNA Kit (TIANGEN). cDNA synthesized through a PrimeScript RT reagent Kit with gDNA Eraser (TaKaRa) and using random hexamers as primers (42 °C for 2 min, 37 °C for 15 min, 85 °C for 5 s). Forward primer GACAAGAGCAAACGGTTGAACAC and reverse primer GCCCAGAGCCTCTCATTGTAG were used for the qRT-PCR for ACE2. Genes were analyzed using Realstar Green Fast Mixture UNG with ROX (GenStar A305–05) on a LightCycler 480 Real-Time PCR system (Roche). Internal H1299 mRNA levels were used for normalization and the fold expression was calculated using the 2^−ΔCT^ method compared to the 293 T, Huh7, HepG2, H1299, H460, U251, MRC5 cells mRNA levels.

### Western Blotting

HEK293T, Huh7, HepG2, H1299, H460, U251 and MRC5 cells were harvested, washed with PBS and subsequently lysed in RIPA Lysis and Extraction Buffer (Thermo Fisher). Total protein levels were determined by a bicinchoninic acid assay (ThermoFisher), and 10 μg of protein was resolved by 12% SDS polyacrylamide gel electrophoresis. Proteins were transferred to nitrocellulose, blocked in 5% bovine serum albumin (BSA), and incubated with anti-ACE2 antibody (10108-T24, Sino Biological) or anti-β tubulin (sc-166,729; Santa Cruz). Protein was detected using horseradish peroxidase (HRP)-conjugated anti-rabbit secondary antibody (Amersham) and Luminata Forte Western HRP substrate (Millipore).

### Co-IP

HEK293T/ACE2 stable cell line was generated by transducing HEK293T cells with pCMV-Flag-ACE2,(Sino Biological, HG10108-NF), and 1 × 106 cells were lysed in lysis buffer (50 mM Tris-HCl, pH 7.4, 150 mM NaCl, 2 mM EDTA, 1% NP-40, 1% sodium deoxycholate), supplemented with protease inhibitors. Lysates were first incubated with S1 or S1(D614G) for 4 h at 4 °C, then incubated with anti-FLAG antibody (M2, Sigma-Aldrich) or mouse IgG conjugated to Dynabeads Protein G (Thermo Fisher Scientific) overnight at 4 °C. The beads were washed with wash buffer (50 mM Tris-HCl, pH 7.4, 150 mM NaCl, 2 mM EDTA, 1% NP-40, 1% sodium deoxycholate) five times. Eluted protein samples were separated by SDS–polyacrylamide gel electrophoresis, and immunoprecipitated with anti-Flag antibody (Sigma, F7425) or anti-spike antibody (ThermoFisher, PA5–81795).

### Cell Capture and Detached by Immobile S1

96-well ELISA plates (CIH-F8T, GSBIO) were coated with 0.5 μg/well of spike S1-his protein in PBS buffer (pH 7.0) overnight at 4 °C. After washing with 0.05% Tween 20-PBS (w/v), wells were and blocked with 3% BSA-PBS (w/v) for 1 h. For cell capturing, 3 10^4^ cells were added to each S1-coated well, incubated for 15 min at 37 degree, then washed 5 times with PBS (containing 1% BSA). To quantify captured cells, three methods were utilized. The first one is the CCK8 test, where captured cells were washed once with 300 μl PBS, incubated with 100 μl CCK8 working solution (complete medium: CCK8 = 10:1) at 37 °C, 5% CO2 for 3 h. Subsequently, OD values at 450 nm were determined and number of cells was calculated according to the standard curve. Alternatively, cells were detached by using Trypzin-EDTA then counted by flow cytometry. In case number of captured cells was low, captured cells were counted manually under the Optical microscope.

### Quantification of Cell Viability

Cell viability of captured cells was analyzed using the Annexin V-FITC Apoptosis Detection Kit (Beyotime). In brief, the captured cells were digested with trypsin, centrifuged at 300 g for 5 min and washed twice with PBS. The cell pellet was gently resuspended with 195 UL binding solutions, stained with Annexin V-FITC (5ul) and propidium iodide (10 UL) in the dark for 10-20 min, then immediately deterimined using flow cytometry.

### Immunofluorescence Staining

ACE2-GFP transfected HEK293T cell at 70% confluent were washed with PBS, fixed for 10 min with cold 100% methanol, and blocked using 5% BSA in PBS for 2 h. Subsequently, cells were incubated with a 1:2000 diluted anti-ACE2 rabbit polyclonal (10108-T24, Sino Biological) overnight at 4 °C, washed with PBS and incubated for 2 h with Alexa 488-conjugated goat anti-rabbit IgG antibody (Invitrogen). After staining, slides were mounted using ProLong Antifade (Thermo Fisher) solution and observed using a Leica SP8 confocal microscope.

### Mice Anti-Spike Serum Sample Preparation

Eight-week-old female BALB/c mice were obtained from Weitonglihua Co. Limited (Beijing, China) and housed in a specific pathogen-free (SPF) animal facility. Mice were immunized with 50 μg of SARS-Cov-2 spike protein (40607-V08B, Sino Biological) emulsified incomplete Freund’s adjuvant (CFA) by subcutaneous injection of up to 100 μl volume per site. Two and 4 weeks after the first immunization, mice were boosted another with S protein emulsified in Incomplete Freund’s adjuvant (IFA). Serum samples from immunized mice or control mice were prepared and tested by ELISA with a minimum dilution of 100-fold to confirm antibody response.

### Statistical Analysis

Results shown in each figure were derived from at least three independent experiments with comparable findings. The comparison of means between two groups was conducted using Student’s t-test, whereas comparison for more than two groups was conducted using one-way ANOVA. Only *p* values of 0.05 or lower were considered statistically significant. All statistical analyses were performed using the GraphPad Prism 7 software package (GraphPad Software).

## Supplementary Information

**Additional file 1.**

## Data Availability

All data generated or analyzed during this study are included in this published article.

## Abbreviations

SARS-CoV-2: Severe acute respiratory syndrome coronavirus 2
ACE2: Angiotensin converting enzyme 2
S protein: SARS-CoV-2 spike protein
S1 protein: s1 domain of SARS-CoV-2 spike protein
COVID-19: Coronavirus disease 2019
CCK8: Cell counting kit-8
RBD: Receptor-binding domain
IC50: Half-maximal inhibitory concentration
HEK293T: Human embryonic kidney 293 T cell line
H1299: NCI-H1299 cell line (human lung adenocarcinoma cells)
H460: NCI-H460 Cells (Lung carcinoma cells)
HUH7: HUH-7 cells (human liver cancer cells)
HepG2: HepG2 cell line (human hepatocellular carcinoma cells)
MRC5: MRC-5 cell line (human fetal lung fibroblast cells )
U251: U251 cell line (human glioblastoma cell)
