# Supplementary material for: Investigation of Interaction between the Spike Protein of SARS-CoV-2 and ACE2-Expressing Cells Using an In Vitro Cell Capturing System
Source: Biol Proced Online. 2021 Aug 26;23:16. doi: 10.1186/s12575-021-00153-9 (PMC8387204; doi:10.1186/s12575-021-00153-9)
Supplement: Supplementary file 1 — Additional file 1. [file 12575_2021_153_MOESM1_ESM.docx]

**
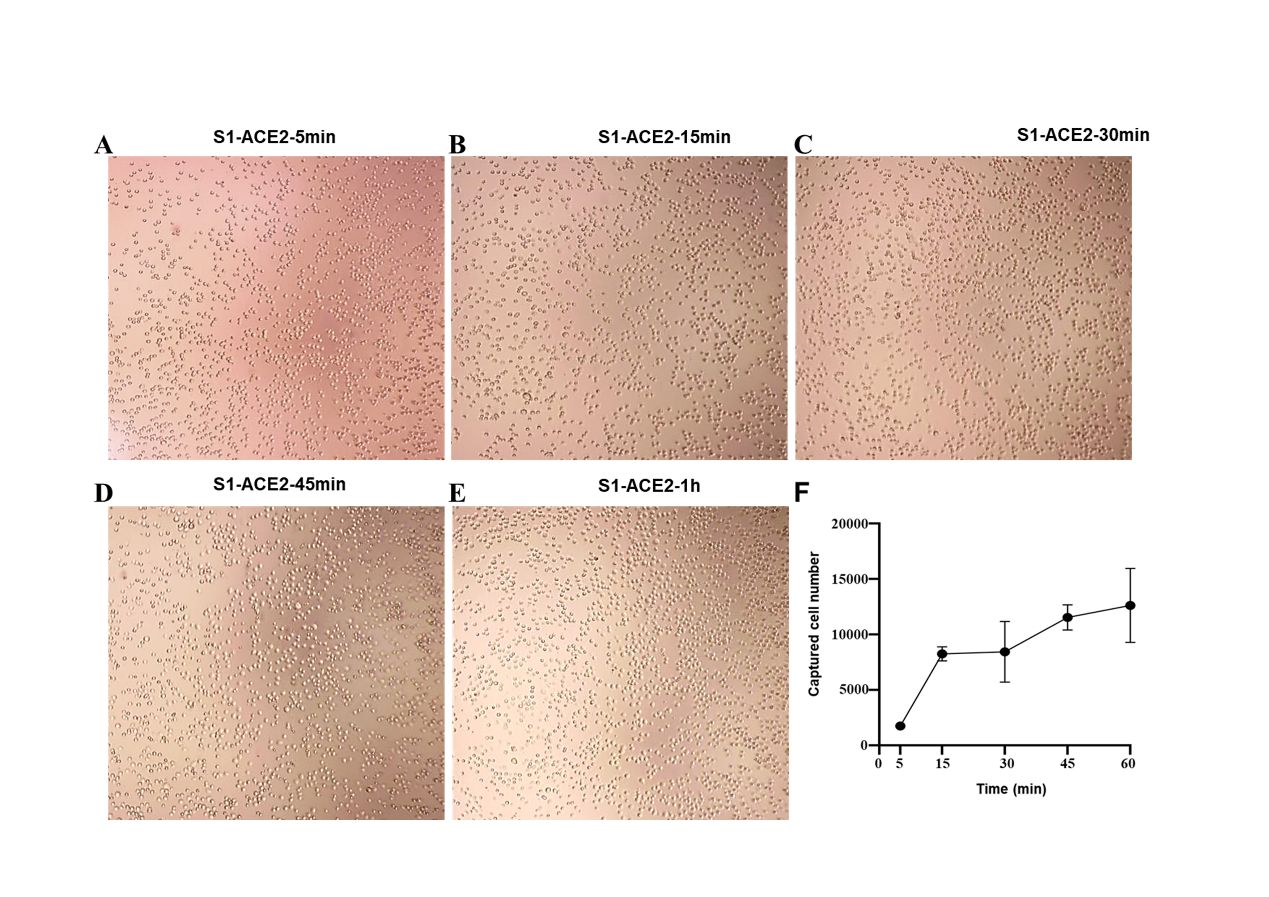
Suppl. Fig. 1: Cell capturing kinetics of immobilized spike protein.** Time kinetics of the captured cells number by immobilized spike protein. Photograph the cells of different binding time as A-to-E, and (F) kinetic curve of cells number via binding time course. Quantitation data are presented as mean ± SD.
